# Removal of Dyes Using Graphene Oxide (GO) Mixed Matrix Membranes

**DOI:** 10.3390/membranes10120366

**Published:** 2020-11-25

**Authors:** Rana J. Kadhim, Faris H. Al-Ani, Muayad Al-shaeli, Qusay F. Alsalhy, Alberto Figoli

**Affiliations:** 1 Civil Engineering Department, University of Technology, Alsinaa Street 52, Baghdad 10066, Iraq; rana1979.kadhim@gmail.com (R.J.K.); 40027@uotechnology.edu.iq (F.H.A.-A.); 2 Department of Chemical Engineering, Monash University, Clayton, VIC 3800, Australia; muayad.al-shaeli@monash.edu; 3 Membrane Technology Research Unit, Chemical Engineering Department, University of Technology, Alsinaa Street 52, Baghdad 10066, Iraq; 4 Institute on Membrane Technology, National Research Council (ITM-CNR), 87030 Rende, Italy; a.figoli@itm.cnr.it

**Keywords:** PES membrane, graphene oxide, hydrophilicity, membrane formation, wastewater treatment, polyvinylpyrrolidone

## Abstract

The application of membrane technology to remove pollutant dyes in industrial wastewater is a significant development today. The modification of membranes to improve their properties has been shown to improve the permeation flux and removal efficiency of the membrane. Therefore, in this work, graphene oxide nanoparticles (GO-NPs) were used to modify the polyethersulfone (PES) membrane and prepare mixed matrix membranes (MMMs). This research is dedicated to using two types of very toxic dyes (Acid Black and Rose Bengal) to study the effect of GO on PES performance. The performance and antifouling properties of the new modified membrane were studied using the following: FTIR, SEM, AFM, water permeation flux, dye removal and fouling, and by investigating the influence of GO-NPs on the structure. After adding 0.5 wt% of GO, the contact angle was the lowest (39.21°) and the permeable flux of the membrane was the highest. The performance of the ultrafiltration (UF) membrane displayed a rejection rate higher than 99% for both dyes. The membranes showed the highest antifouling property at a GO concentration of 0.5 wt%. The long-term operation of the membrane fabricated from 0.5 wt% GO using two dyes improved greatly over 26 d from 14 d for the control membrane, therefore higher flux can be preserved.

## 1. Introduction

As a result of the huge discharge of industrial wastewater and the shortage of water resources, the accumulation of waste and contaminants in water is a global environmental problem leading to unsafe water for human use. Additionally, the global demand for fresh water has increased significantly due to population growth, increasing urbanization, industrialization, and expanding economies. Therefore, sustainable and clean technologies are urgently required to solve this global challenge [1].

Due to the high salinity and toxicity of azo dyes, they are one of the most dangerous pollutants in wastewater [2,3]. They play a key role in coloring cellulose fibers (such as cotton), and the use of reactive dyes can cause serious environmental problems [4]. Due to the complexity of dye molecules and their resistance to aerobic digestion, oxidants, light, and heat, wastewater from the textile industry is difficult to treat [5]. The main industries that release dyes into the environment are the textile industry, which creates over half (54%) of the existing dye wastewater worldwide; dyeing industry (21%); pulp and paper industry (10%); paint and leather industry (8%); and dye manufacturing industry (7%) [6]. Therefore, it is critical to use clean, economical, and effective treatment methods, such as membrane separation, to treat dyes.

Membrane separation technologies are considered to be the best solution to overcome water scarcity by providing high-quality fresh water to many people. These technologies boast outstanding properties, such as low operating costs, high separation efficiency, reliability, simplicity, and being more environmentally friendly than conventional water treatment technologies. Therefore, these methods have been applied in many industries, including waste treatment [7], pharmaceutical synthesis [8], food manufacturing, dyestuff industry [9], brackish water treatment, and desalination [10].

As a part of membrane separation technology, the use of an ultrafiltration (UF) membrane is a clean, efficient, and attractive technology, which effectively removes suspended particles, bacteria, and organic compounds. Polymeric materials used to fabricate ultrafiltration membranes include polyethersulfone (PES), polyvinylidene fluoride (PVDF), polyacrylonitrile (PAN), and bromomethylated polyphenylene oxide (BPPO). Although these polymer materials are commercially available, their low permeation flux and fouling issues limit their applicability in industry. Researchers have devised many methods to improve the performance of polymeric membranes, including the use of polymer blends [11]; various additives, including water-soluble materials [12], nanoparticles, and nanomaterials [13,14,15,16,17,18,19]; and free radical graft copolymerization (chemical modification) [20]. Among the several polymeric materials used to manufacture ultrafiltration membranes, significant interest lies with polyethersulfone membranes (PES) due to the beneficial properties of PES, including excellent chemical and thermal stability, remarkable oxidation resistance, outstanding robustness and tolerance of solvents, and enhanced mechanical properties. One of the main obstacles to PES membrane use is fouling, which reduces the membrane flux either permanently or temporarily, thus affecting the lifetime of the membrane. In fact, a PES membrane is influenced by fouling problems because of the interactions between the surface charges of the PES membrane and the foulants, which may be organic, inorganic, or biological forms. Therefore, the hydrophilic modification of PES is a crucial task, which can provide higher separation efficiency and higher antifouling performance to durable UF membranes for high-quality water treatment. 

As mentioned above, many research studies have used hydrophilic nanomaterials and nanoparticles as nanofillers blended with polymer materials to enhance the hydrophilicity and antifouling performance of the UF membranes. However, within the past seven years, many studies have focused on using graphene oxide nanoparticles (GO-NP_S_) as a potential for dye removal due to its exceptional conjugated two-dimensional structure (2D), which has demonstrated a higher adsorption capacity for various dye molecules through π–π stacking interactions [21,22,23,24,25,26]. Moreover, the negative charges in the GO sheets that result from various oxygen-rich functional groups (i.e., carboxyl (-COOH), carbonyl, epoxy (C-O-C), and hydroxyl groups (-OH)) allow for additional strong electrostatic interactions with cationic dye molecules [26,27,28]. The incorporation of GO sheets into the polymer appears to make it highly attractive for multiple purposes due to its remarkable properties. These include a two-dimensional structure, the ability to promote negative surface charges, outstanding electron transport, high surface area, innocuity, and remarkable chemical stabilities. GO can also change the roughness and mechanical properties of membranes and the effects of membrane fouling. It can increase water permeation flux and surface hydrophilicity, improve mechanical strength, reduce both organic and biofouling propensity, and efficiently separate dye [29]. 

Among the GO studies in membrane technology, Zinadini et al. [30] investigated the impact of blending a GO nanoplate on PES/ polyvinylpyrrolidone (PVP) nanofiltration membranes for dye removal and powder milk filtration. GO-modified PES membranes exhibited a higher dye removal capacity than pristine PES-UF membranes. Wang et al. [31] found that a GO nanosheet blended into a PVDF polymer matrix improved the membrane hydrophilicity, water permeation flux, and antifouling performance of the membrane. Other researchers used GO as a filler in different separation applications to modify polymeric membranes (e.g., PES, polysulfone (PSF), and PVDF membranes) [32,33,34,35,36,37,38,39,40] and make mixed matrix membranes (MMMs). They proved that GO is a great candidate to enhance the membrane water flux and keep/maintain the % rejection at a high level. 

The aim of this work is to prepare mixed matrix membranes (MMMs) with higher capabilities to remove very toxic dyes from wastewater. Modifying a PES membrane for the removal of very toxic dyes such as Acid Black-210 and Rose Bengal, which are dissolved in wastewater of the leather tanning and textile industries, respectively, has not been investigated in detail. In fact, wastewater of the leather tanning and textile industries is a complex mixture and unmodified membranes cannot treat it and remove such dyes effectively. Therefore, a favorable technique for modifying a PES membrane with GO for removal of Acid Black-210 and Rose Bengal is necessary and workable. Moreover, the effect of embedded GO in the polymer solution on the cross-sectional structure of the membrane was not presented previously in detail. Therefore, in the current research study, GO particles were added to PES UF casting solutions for the removal of dyes from wastewater of leather tanning and textile industries. The effect of GO on the cross-sectional morphology and hydrophilicity was extensively investigated. The efficiency of the PES membranes was measured in terms of the permeation flux and the anionic dye removal efficiency of Acid Black-210 and Rose Bengal. The synthesized GO and the prepared membrane were characterized by Fourier transform infrared (FTIR) spectroscopy, scanning electron microscopy (SEM), atomic force microscopy (AFM), and water contact angle (CA) measurement.

## 2. Experimental Work

### 2.1. Chemicals

All the chemicals used in the experimental work were of a reagent grade. Polyethersulfone polymer (PES; Ultrason E6020P, 51 kDa) was purchased from Solvay Specialty Polymers, Belgium. Polyvinylpyrrolidone (PVP; 25 kDa) was provided by Mowiol, Germany. Dimethyl sulfoxide (DMSO; (CH_3_)_2_SO) with a density of 1.1 g/mL and Mwt = 78.13 g/mol was utilized as a polymer solvent and obtained from Sigma-Aldrich (St. Louis, MO, USA). Sulfuric acid (H_2_SO_4_; 98%) was provided from Central Drug House, Delhi, India. Potassium persulfate (K_2_S_2_O_8_) was ordered from RIEDEL-DE HAEN AG, Seelze-Hannover. Phosphorus pentoxide (P_2_O_5_; white powder, Mwt = 141.96) was purchased from Scharlau, Australia. Graphene platelet powder (6–8 nm pore size) was ordered from Skyspring Nanomaterials, Inc. (Houston, TX, USA). Acid Black 210 dye (C_34_H_25_N_11_O_11_S_3_; Mw = 938.017g/mol) was procured from Leeds, United Kingdom. Rose Bengal dye (C_20_ H_2_ CL_4_ L_4_ Na_2_ O_5_; Mw = 1017 g/mol, λ_max_ = 520 nm) was purchased from HiMedia Laboratories, India.

### 2.2. Preparation of the Graphene Oxide (GO)

Graphene oxide (GO) was synthesized using a modified Hummers method. First, a solution was prepared that consisted of 120 cm^3^ concentrated sulfuric acid (H_2_SO_4_), 15.6 g potassium persulfate (K_2_S_2_O_8_), and 16.8 g phosphorus pentoxide (P_2_O_5_). Then, 30 g of graphite was added to the prepared solution and refluxed in a condenser at 75–80 °C for 4 h. Next, the solution was allowed to cool at ambient temperature. After that, the solution was filtered and washed successively with distilled water until the pH became neutral (i.e., pH = 7). The product was dried in an oven at 60 °C for 48 h [18]. 

### 2.3. Preparation of the Membrane

To further prepare the membrane, dried PES powder (21 and 1 wt% PVP) was dissolved in dimethyl sulfoxide (DMSO) solvent and stirred using a magnetic stirrer for 24 h at ambient temperature. PVP was used as the pore-forming additive. GO particles at different concentrations from 0.1 to 2 wt% were added to the homogeneous PES–PVP solution and stirred for more than 3 h. Then, the PES–PVP–GO solutions were kept in an ultrasonic bath (operated at 50–100 watts per gallon) for 30 min to avoid the agglomeration of the GO-NPs in the final casting solution. Then, the casting solution was poured on a precleaned glass plate, and a self-made casting knife was used to cast at room temperature. The phase inversion was carried out by soaking the casting solution in a water coagulation bath for 24 h. The membrane was then taken from the bath and rinsed thoroughly with double deionized water (DDI) and stored in fresh DDI before use. Next, the membrane was placed in another water bath mixed with glycerin to prevent the membrane structure from collapsing. Table 1 lists the composition of the casting solution for all of the membranes.

### 2.4. Membrane Characteristics

#### 2.4.1. Fourier Transform Infrared (FTIR) Spectroscopy

A Fourier transform infrared-attenuated total reflection (FTIR-ATR) spectrometer (PerkinElmer, Australia) was used to check the chemical structure of the membrane. It was characterized by FTIR with a resolution of 4 cm^−1^ in the range of 500–4000 cm^−1^, and the average value was taken over 64 scans.

#### 2.4.2. Scanning Electron Microscopy (SEM)

A scanning electron microscope (SEM) measurement (TESCAN VEGA3 SB instrument, EO Elektronen-Optik-Service GmbH, Germany) was used to examine the top membrane surface and cross-sectional morphology of the control membrane and the modified PES membrane at an accelerating voltage of 30 KV. Membrane samples were prepared by drying the membrane at room temperature and sputtering the coating with a 0.5-nm thickness of Pd metal. For cross-sectional images, the membrane was broken in liquid nitrogen to retain the membrane structure and then, inspected using SEM equipment. The cross-section and outer surface of the membrane were scanned at several magnifications. The PES/GO membrane was analyzed using SEM and an energy dispersive X-ray spectrometer (SEM-EDX). After the SEM images were taken, analysis of the GO-NPs by EDX was conducted.

#### 2.4.3. Atomic Force Microscopy (AFM)

A scanning probe microscope (SPM AA300 Angstrom Advanced Inc., AFM, USA) was used to characterize the surface morphology and roughness of the prepared membrane. A membrane sample was cut about 1 cm^2^ and fixed on the holder, where an 8.8 × 8.8 µm area was scanned. The surface roughness parameter is reflected as the average roughness S_a_, the root mean square of the Z data S_q_, and the average difference between the highest peak and the lowest valley S_z_.

#### 2.4.4. Contact Angle 

The hydrophilicity of the membrane was measured using a contact angle goniometer by the sessile drop technique (110-O4W CAM, Tainan, Taiwan). To achieve optimal accuracy, at least five contact angle measurements at different locations for each membrane sample were recorded, and an average was calculated.

#### 2.4.5. Porosity

Using the gravimetric method, the porosity of the membranes was determined, as defined in the following equation [41]:

(1) $$\varepsilon =\frac{W1-W2\;}{A*T*\;\rho }.$$

where ε is the porosity (%); W1 is the weight of the wet membrane (g); W2 is the weight of the dry sample (g); A is the membrane effective area (cm^2^); T is the membrane thickness (μm); and ρ is the water density (0.998 g/cm^3^ at 25 °C). The membrane samples of pristine and modified PES membranes were soaked in distilled water for 24 h at room temperature. Then, any water was wiped off the membranes, and each membrane was weighed [41].

#### 2.4.6. Pore Radius 

The mean radius of the membrane pore (rm) was estimated by Equation (2) (i.e., the Guerout–Elford–Ferry equation) [39].

(2) $$r_{m}=\sqrt{\frac{\left(2.9-1.75\;\varepsilon \right)8\eta \;l\;Q}{\varepsilon \times A\times \;\Delta P}}$$

where r_m_ is the mean pore radius (nm), η is the viscosity of the water (8.9 × 10^−4^ Pa s), l is the thickness of the membrane (m), Q is the collected volume of the permeate per unit time (m^3^/s), A is the membrane surface area (m^2^), and ΔP is the transmembrane pressure (Pa).

#### 2.4.7. Mechanical Properties (Tensile Strength)

A Zwick/Roell device (Germany) was used for the tensile measurement of the membrane at room temperature. The membrane sample was cut into 10 × 100 mm shapes. The three samples were checked to ensure they had a loading rate of 5 mm/min for breaking strength, and the average of the tensile strength was calculated to increase the accuracy. The tensile strength at the fracture was calculated by dividing the force at the fracture (N) by the cross-sectional area of the sample (m^2^) [42].

#### 2.4.8. Zeta Potential Test

A zeta potential analyzer (Zetasizer 3000HS, Malvern Co., UK) was used to determine the zeta potential of the organic dye within a wide pH range. Initially, the dye was dried in an oven at 50 °C to remove water, after which it was dissolved in deionized water. Sodium hydroxide and hydrochloric acid were used to adjust the pH of the dye solution to be in the range from 3 to 10. At least three rounds were performed for each sample, and the average value was recorded. 

#### 2.4.9. Membrane Filtration Experiments

**Figure 1:**
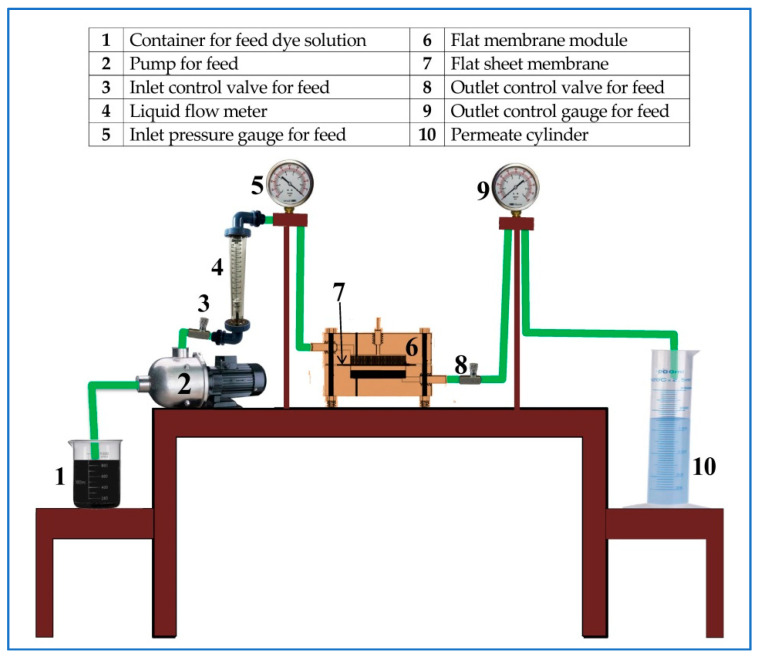
Schematic diagram of the cross-flow filtration rig.

The pure water flux (PWF) of the pristine and PES–PVP–GO membranes was measured using the cross-flow filtration process shown in Figure 1. The effective membrane filtration area was 16 cm^2^. Initially, each membrane sample was pre-compacted for at least 90 min until the flux stabilized at 3 bar. Then, filtration tests were conducted at a steady pressure of 3 bar and temperature of 25 ± 1 °C. 

The rejection test was performed with four aqueous solutions (i.e., 10, 50, 80, and 100 ppm) of Acid Black-210 and Rose Bengal dyes. The pH value of each of the two dye solutions naturally was equal to 5. To calculate the separation efficiency of the membranes, the concentration of dyes in the permeate solution was determined using a spectrophotometer (UV 1100) at λ_max_ of 460 and 520 nm. The pure water flux, *J* (L/m^2^.h) and dye separation efficiency *R* (%) were determined according to Equations (3) and (4), respectively.

(3) $$J_{1}=\frac{V}{A*t}$$

where *J*_1_ is the pure water permeation flux of the membrane (L m^−2^ h^−1^); *V* is the volume of the permeate collected (L); *t* is the time the permeate was collected (h); and *A* is the membrane surface area (m^2^).

(4) $$\%\;R=\left(1-\frac{C_{f}}{C_{p}}\right)\times 100$$

where *C_f_* and *C*_0_ are the concentration of the dye in the feed solution and the permeate (mg/L), respectively.

After testing, the fouled membranes were washed with water three times for 30 min, and the pure water flux of the washed membranes (*Jw_2_*, L/m^2^ h) was recorded. The flux recovery ratio (% *FRR*) was calculated by comparing the flux after each cleaning cycle to the pure water flux. The following equation was used to compare the fouling resistance of the fabricated membranes [43]:

(5) $$\%\;FRR=\;\frac{J_{2}}{J_{1}}\times 100$$

where *J*_1_ and *J*_2_ are the flux (L/m^2^ h) before and after the filtration test, respectively. 

## 3. Results and Discussion

### 3.1. Characterization of the Composite Membranes

#### 3.1.1. FTIR-ATR Spectrum Results 

**Figure 2:**
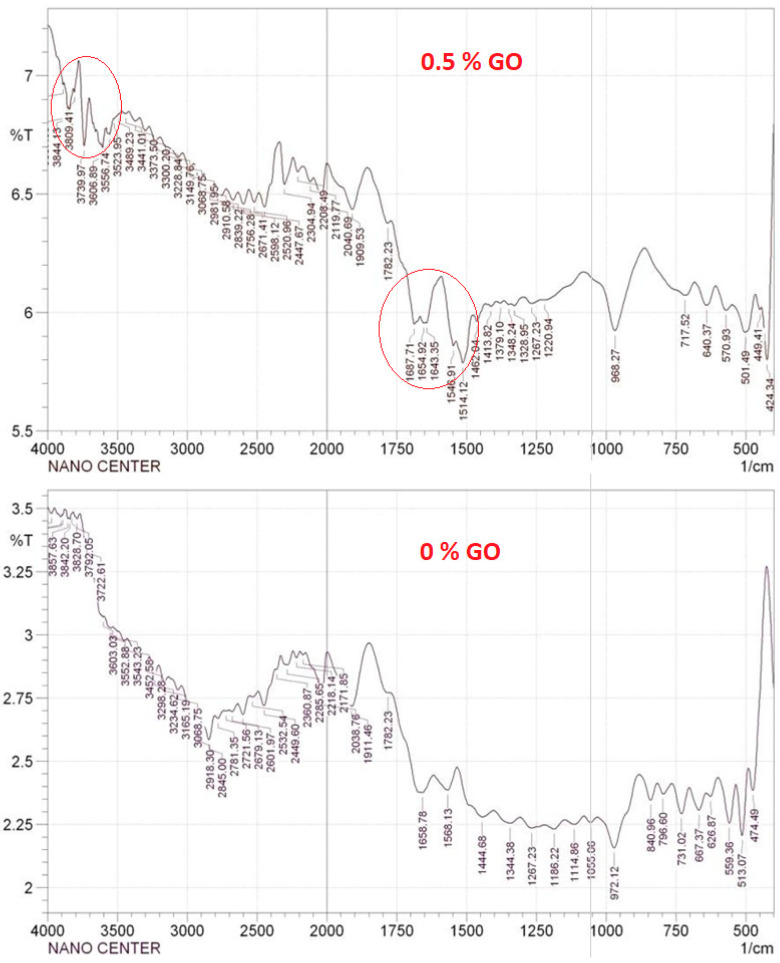
Fourier transform infrared-attenuated total reflection (FTIR-ATR) spectrum of the prepared membranes.

FTIR-ATR was performed to check the surface compositions of the GO and to compare the surface composition of the PES and GO-modified PES membranes. The FTIR results of the control and modified PES membranes are shown in Figure 2. The spectral peaks located at 1735, 1626, 1248, and 1050 cm^−1^ are attributed to the stretching vibration of the carbonyl group -C=O, the stretching vibration of the unoxidized graphitic domain C=C, and the stretching of the epoxy groups C-O-C and C-OH, respectively. The absorption peaks at the wavelengths of 3414, 1556, and 1120 cm^−1^ in the GO nanoparticles correspond to the functional groups of hydroxyl (-OH) and carboxyl (-C=O and C-O), respectively. FTIR affirmed the existence of different oxygen-containing functional groups, such as hydroxyl, carboxyl, carbonyl, and epoxy. These functional groups can change the membrane structure by improving its hydrophilicity [30]. The spectra also show two bands at 1078 and 1234 cm^−1^ that were caused by the stretching vibration of the alkoxyl C=O. The control PES membranes presented characteristic bands at 1250, 1486, and 1578 cm^−1^ that belong to the stretching vibrations of C-O-C, aromatic bands, and the benzene ring, respectively. The peak between the range of 1290–1325 cm^−1^ was due to O=S=O [44]. 

After the GO-NPs were introduced into the PES membrane (0.5 % GO), the bands at 1627 and 1690 cm^−1^ were assigned to the vibration of the adsorbed water molecules and the contribution of the aromatic C=C vibration. As shown in Figure 2, the FTIR spectra were used to confirm the generation of functional groups. Due to the asymmetric and symmetrical extension of the SO_2_ group, there was bonding at 1320 and 1149 cm^−1^. The peaks at 3097 and 3071 cm^−1^ corresponded to the -OH group of the carboxylic acid functional groups that are present in the GO-NPs and aromatic C-H bond stretching, respectively. The peak at 1103.94 cm^−1^ was attributed to the C-O stretching bond, and the C-O-C bond was represented by the peak at 1239.13 cm^−1^. The peak at 2955 cm^−1^ corresponds to the asymmetric stretching of CH_2_ in the PVP.

#### 3.1.2. Morphology Examination by Scanning Electron Microscopy (SEM)

**Figure 3:**
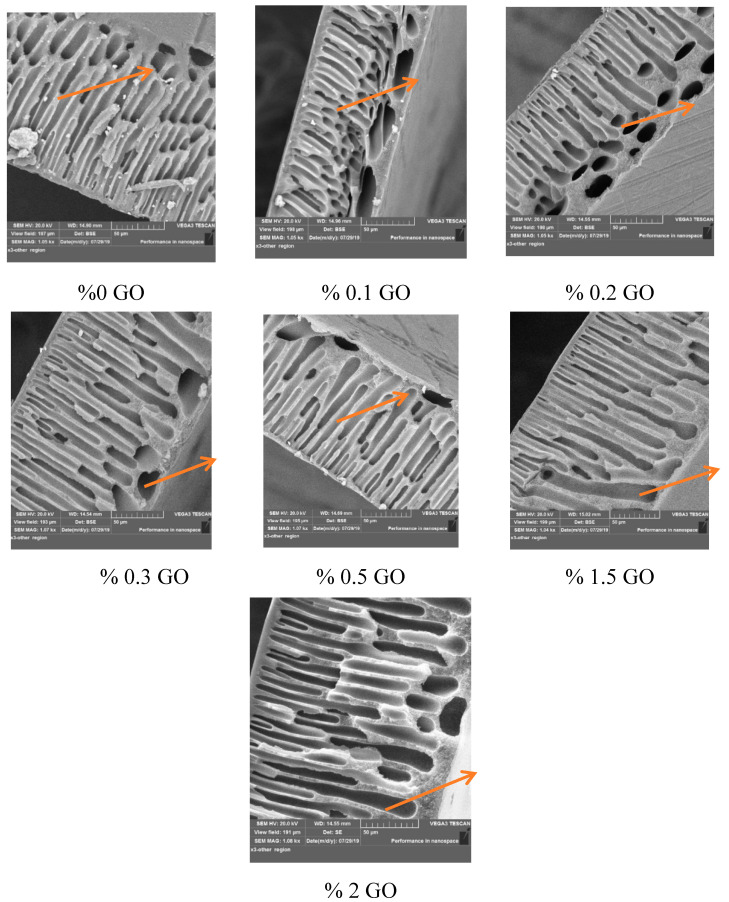
Cross-section SEM images of the control and GO-modified PES membranes.

Figure 3 shows cross-sectional images of the control and synthetic PES membrane with different GO contents. All the synthetic PES membranes exhibited typical symmetrical, porous structure characteristics, with the structure composed of two layers. A large layer appeared near the top edge of the membrane, with a long and thin finger-like structure. Additionally, a small layer appeared near the edge of the bottom surface, consisting of a sponge-like structure and many elliptical voids. The embedding of the GO into the casting solution at different concentrations altered the thin finger-like structure gradually into a wide finger-like structure extending to the edge of the bottom surface. The colored arrows in Figure 3 illustrate the stages of impact of the embedding of different GO amounts in the polymeric solution on the cross-sectional structure of the composite membranes. This phenomenon is attributed to the fact that GO, as a hydrophilic medium, accelerates the exchange rate between the solvent (DMSO) and nonsolvent (water as the coagulation media) during the phase inversion process, resulting in the formation of larger pores [45]. Due to the presence of hydrophilic functional groups, the migration of GO to the membrane surface will enhance the hydrophilicity of the membrane [30], which was confirmed by the FTIR-ATR results.

Figure S1 (Supplementary Information) displays the top surface images of the control and GO/PES composite membranes. As shown in Figure S1, the surface of the membrane was relatively smooth, and no pores were observed for all the prepared membranes. This phenomenon fits with our objective that the addition of the GO to the PES casting solution would accelerate the exchange rate between the solvent and nonsolvent (DMSO–water) during nascent membrane formation at a high polymer concentration in the casting solution. Therefore, a smooth-skinned, selective layer formed [43]. 

The elemental composition for the control and modified nanocomposite PES membranes was verified by the EDX analysis. Figure S2 illustrates the distribution of the GO taken from the top surface of the PES membrane. It can be seen that by adding different concentrations of GO-NP to the casting solution, the dispersion of the GO-NP (carbon element) in the membrane matrix improved. Most importantly, as the loading of the GO-NPs into the PES–PVP polymer casting solution increased, the C in the membrane matrix had a larger agglomeration. This was due to the excellent dispersibility and homogeneity of the GO in the casting solution as a result of using an ultrasonic device.

#### 3.1.3. Atomic Force Microscopy (AFM) and Mean Pore Radius 

Figure S3 shows the 3D atomic force microscope (AFM) measurement of the control PES and GO/PES composite membranes in the 8000 × 8000 nm scanning area. In these pictures, the darker areas represent valleys or membrane pores, while the brightest areas represent the highest points on the membrane surface (e.g., nodules). The nodules of the control PES were large and connected with one another on the 3D surface. In addition, adding 0.1% by weight of GO to the PES casting solution resulted in an increase in the number of nodules. As shown in Figure S3C–E, as the GO in the casting solution further increased to 0.5% by weight, the number of nodules increased with a decrement in their size at the membrane surface. When the content of the GO was increased from 1.5% to 2% by weight, the nodules were uniformly shaped, and they were also uniformly distributed on the membrane surface, as shown in Figure S3F,G. Nodule size and density are good indicators of pore size and pore distribution [19]. Table 2 shows the effect of GO content on mean roughness and the mean pore radius of the control PES and GO/PES membranes. 

It can be noticed that there is no clear trend of the effect of GO on membrane roughness. In general, it can be concluded that the mean roughness is decreased with the addition of GO in the casting solution. The mean pore radius of the membrane was highly enhanced from 8.25 nm for the control PES to 14.59 nm when the concentration of GO was 0.5 wt%. With further increases in the amount of GO (1.5 wt%), the mean pore radius of the composite membranes further increased to 15.59 nm, while a slight decrease was observed with further increases in the amount of GO to 2 wt%. This occurred due to the high hydrophilic character of GO for water, which in turn accelerated the exchange rate between the DMSO solvent and the nonsolvent water (e.g., instantaneous liquid/liquid demixing process) [46].

#### 3.1.4. Thickness and Porosity of Pristine PES and PES–PVP–GO Membranes

**Figure 4:**
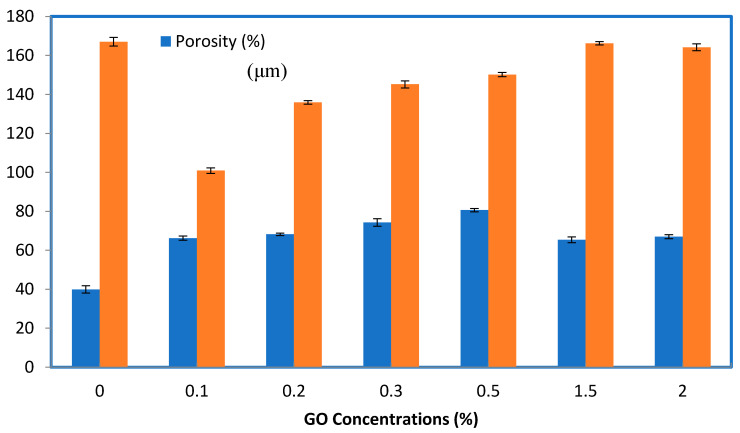
Effect of GO concentrations on the thickness and porosity of PES-based membranes. The bar height is the average of five measurements of porosity and thickness and the error bars represent ± one standard deviation.

The thickness of the membrane is a key parameter that affects the membrane’s performance in all membrane separation processes because the membrane thickness acts as a resistance against the mass transfer of compounds within the membrane wall. To enhance the permeation flux of the membrane in applications of ultrafiltration, the membrane must be optimally thin while taking into account the membrane’s mechanical properties. In the current study, the thickness of the membranes was measured using the SEM technique, as depicted in Figure 4. The thickness of the membrane decreased from 167 to 100.88 µm when 0.1 wt% of the GO particles was added to the casting solution. Then, the thickness increased with the addition of 0.2, 0.3, 0.5, and 1.5 wt% of GO amounts to 135.89, 145.16, 150.13, and 166.21 µm, respectively. As the content of the GO particles increased to 2% by weight led to a membrane structure similar to that of the net PES membrane, as shown in Figure 3, the membrane thickness did not decrease significantly. These results were due to the increase in the viscosity of the dope solution with the increasing of the concentration of GO-NPs in the casting solution. The thickness of the membrane was greatly enhanced by embedding the GO particles in the casting solution of the PES–PVP. This occurred due to the affinity of GO for water, which in turn accelerated the exchange rate between the DMSO solvent and the nonsolvent water [46].

Membrane porosity is also a critical parameter that affects membrane performance. The porosity of the membranes for the control PES and GO/PES membranes at different concentrations of GO is displayed in Figure 4. The porosity of the membrane was highly enhanced from 39.92% for the control PES to 80.6% when the concentration of GO was 0.5 wt%. With further increases in the amount of GO (1.5 and 2 wt%), the porosity of the composite membranes declined significantly to 65.4% and 67%, respectively. These results are comparable to previous studies, where an increase in the content of hydrophilic additives led to larger void sizes and higher porosity [47]. The SEM results confirmed the measurements of the membrane thickness and porosity. Figure 4 also shows the effect of GO-NPs concentrations of the thickness of PES membranes. At lower concentrations of GO-NPs in the casting solution, the demixing process becomes faster, which in turn leads to a decrease in membrane thickness, whereas on the other hand, at higher concentrations of GO-NPs in the casting solution, the demixing process becomes slower as the concentration or content of the solid material is increased within the membrane, which leads to an increase in membrane thickness.

#### 3.1.5. Hydrophilicity of the PES and GO/PES Composite Membranes

**Figure 5:**
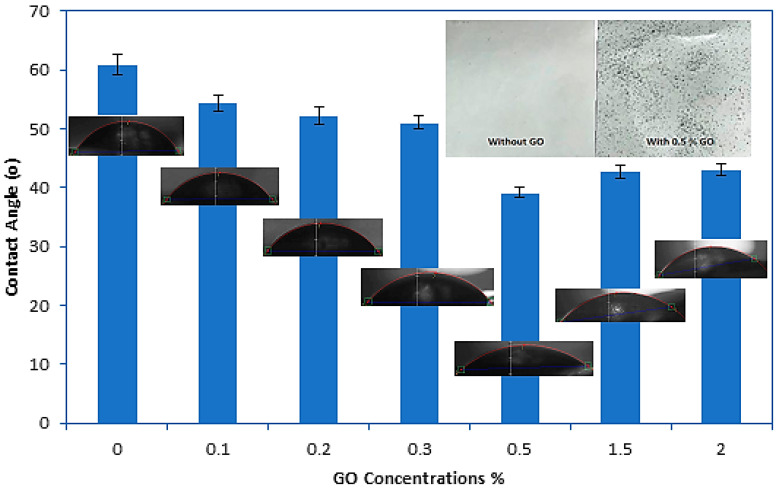
Contact angle measurements for control and GO-modified PES membranes with their top surface photos. The bar height is the average of five contact angle measurements and the error bars represent ± one standard deviation.

The water contact angle of the membrane is a good indicator of the hydrophilicity of the membrane. A lower contact angle indicates that the membrane surface is more hydrophilic. The contact angle results of the control PES and modified GO/PES membranes are shown in Figure 5. As observed in Figure 5, when the GO nanoparticles were added to the polymer matrix, the water contact angle decreased significantly. The control PES showed the highest water contact angle (60.82°). Adding 0.5% by weight to the casting solution reduced the water contact angle to 39.21°, signifying that the membrane surface is hydrophilic. After adding higher concentrations of GO (1.5 and 2 wt%), the contact angle increased slightly, with no significant effect in reducing the hydrophilicity of the membrane. This may be due to the aggregation of GO and the reduction in the effective surface of the particles at high mixing ratios, which in turn led to a decrease in the number of GO functional groups on the membrane surface. During the phase inversion process, the blended nanoparticles in the casting solution could migrate to the top surface of the composite membrane to reduce the interfacial energy [45,48], which can be seen in GO composite membranes. The photos of the top surface of the control and the prepared membrane with a concentration of 0.5 wt% GO were displaced in Figure 5. The results showed that the top surface of the composite membrane was darker than the control PES membrane which was white and clear. During the phase inversion process, as the top layer was more exposed to water, the GO particles moved to the top surface of the membrane. This migration supported more GO functional groups on the membrane surface and improved its surface hydrophilicity. Similar results have been reported in the literature for GO embedding in polysulfone membranes (PSF) [49] or carbon nanotubes (CNT) in the matrix of polyethersulfone membranes [44,50].

#### 3.1.6. Mechanical Properties (Tensile Strength)

**Figure 6:**
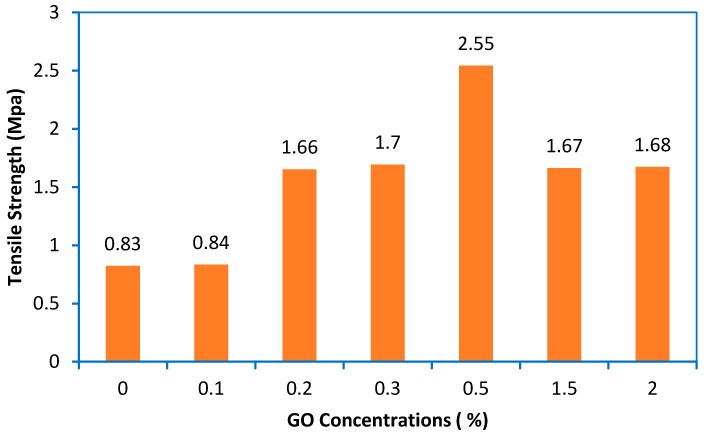
Effect of GO concentrations in casting solution on the tensile strength of the composite membranes.

Figure 6 shows the results of the tensile strength test for the control and modified PES membranes. The membranes showed a gradual improvement in tensile strength from 0.83 MPa for the control PES to 2.55 MPa with GO at 0.5 wt%. At lower concentrations of GO, the tensile strength of the mixed matrix membranes increased and achieved an optimum value at 2.55. The reason might be that a uniform distribution of the GO-NPs was achieved at a rate of 0.5%. This was confirmed by the results of EDX (Figure S2 in the Supplementary Information). Therefore, a concentration of 0.5% is considered as the best addition of GO-NPs to the casting solution. Another reason for obtaining an ultimate strength at 0.5% of GO is that no agglomeration was achieved during the preparation of the casting solution as a lower concentration was used. However, at higher concentrations of GO-NPs, the tensile strength reduced to 1.67 and 1.68 at 1.5 and 2% of GO-NPs, respectively. This is due to a decrease in the movement of the polymer molecules, which increases the resistance of the material to plastic deformation [51].

### 3.2. Performance of Various PES Membranes

#### 3.2.1. Permeation Flux and Dyes Removal

**Figure 7:**
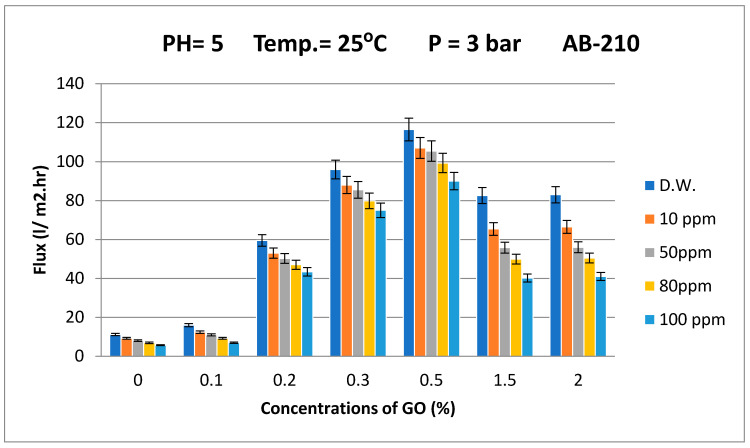
Effect of the Acid Black dye concentration in feed solution on the permeation flux of the membrane prepared at different GO concentrations. The bar height is the average of five measurements of permeation flux and the error bars represent ± one standard deviation.

It has been documented that increasing the surface hydrophilicity will affect the permeation flux of water [30]. Figure 7 shows the results of the permeation flux of the synthesized GO/PES membrane using distilled water (DW) and the simulated wastewater of different concentrations of Acid Black dye (i.e., 10, 50, 80, and 100 ppm) at a pressure of 3 bar and pH = 5, with the temperature of the feed solution 25 ± 1 °C. As shown in Figure 7, the trend of increasing water permeation flux is very consistent with decreasing contact angles (Figure 5). The permeate flux of the membrane increased as the amount of GO particles increased. When the GO content was 0.5 wt%, the maximum water flux was 116.5 LMH because this membrane had the highest porosity, average pore size, and lower contact angle. However, the GO content of 1.5 and 2 wt% led to a decrease in the water flux to 82.3 and 83 LMH, respectively. The decrease in the permeate flux can be attributed to pore blockage caused by the high GO content [52], which indicates a decrease in porosity (Figure 4). The reason for using different concentrations of GO is to study the agglomeration phenomena of nanoparticles within the membranes. In fact, the agglomeration of nanoparticles is a serious problem and would lead to undesirable changes in the properties of the membrane, including lower water flux and weakening membrane mechanical strength. Therefore, to avoid this problem, a lower concentration of nanoparticles is recommended and proposed by many researchers. 

The hydrophilic effect of GO can increase the exchange rate between the solvent and nonsolvent during membrane formation [45]. This leads to higher porosity on the membrane surface (Figure 7) and enhances the permeation flux of water (Figure 7). However, when the GO concentration was higher than 0.5 wt%, the viscosity of the coating solution increased, which led to a decrease in the pore size and the porosity of the membrane [53] and may lead to a decrease in the water permeation flux. A similar behavior was observed by Wang et al. [31] for GO-modified PVDF membranes. 

The permeation flux of Acid Black (AB-210) dye at different concentrations declined moderately, meaning that embedding GO in the membrane matrix minimized the effects of fouling. For example, at a 10-ppm concentration of Acid Black dye, the flux reduction was only 8.12% compared with the pure water flux of the membrane prepared for 0.5 wt% GO, while for the control PES membrane, the flux reduction at 10 ppm of Acid Black dye was 17.85%. These discrepancies can be attributed to the hydrophilicity and antifouling characteristics of the developed membranes. Additionally, it is well-known that increasing the concentration of the dye in the wastewater reduces the permeation flux across the membrane. Therefore, there was a negative effect on the permeation flux when increasing the dye concentration in wastewater of all the control and GO/PES composite membranes. 

From Figure 7, it can be concluded that the results of the permeation flux of the membranes prepared from 1.5 and 2 wt% GO were approximately the same or lower than for the membrane prepared from 0.5 wt% GO. The optimum concentration of GO particles should not be more than 0.5 wt%, which was confirmed by the results of the dye rejection, which will be discussed in the next section.

**Figure 8:**
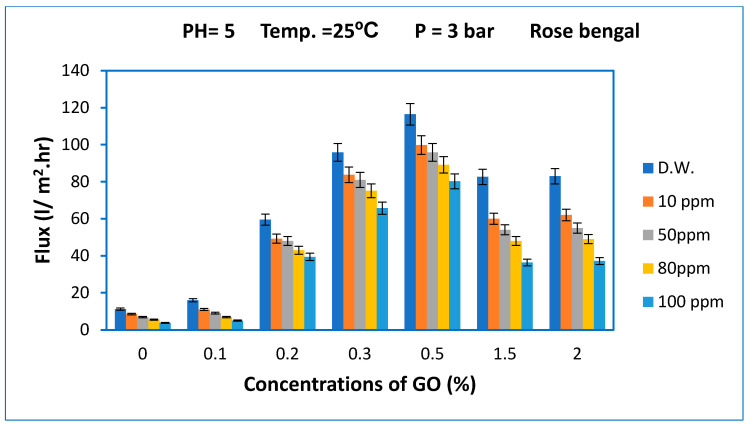
Effect of the Rose Bengal dye concentration in feed solution on the permeation flux of the membrane prepared at different GO concentrations. The bar height is the average of five measurements of permeation flux and the error bars represent ± one standard deviation.

Figure 8 shows the results of the membrane performance of the control PES and GO/PES composite membranes for distilled water and simulated wastewater of different concentrations of Rose Bengal (RB) dye (e.g., 10, 50, 80, and 100 ppm) at a pressure of 3 bar, pH = 5, and feed solution temperature of 25 ± 1 °C. The permeation flux of the control PES and modified PES/GO membranes at various concentrations of Rose Bengal dye were substantially lower than the permeation flux of the Acid Black dye solution. For example, the reduction in the permeation flux at a 10-ppm concentration of Rose Bengal dye was 14.3% compared with the pure water flux of the membrane prepared from 0.5 wt% GO. However, for the pristine PES membrane, the flux reduction at 10 ppm Rose Bengal dye was 23.27%, which represents a higher reduction in the permeation flux than for the pristine PES membrane used for the Acid Black dye.

**Figure 9:**
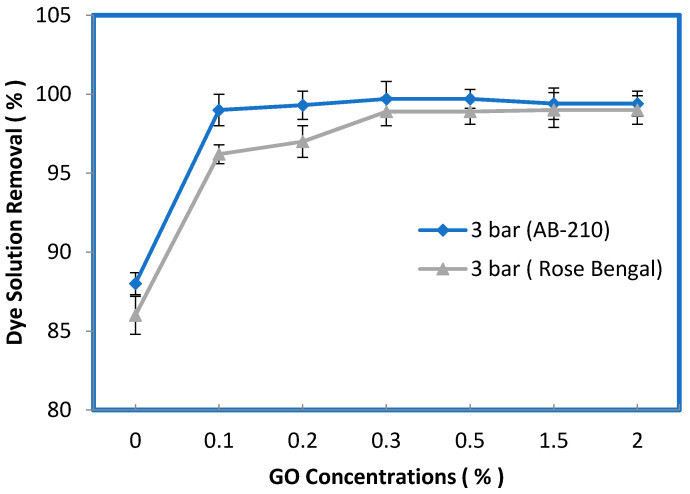
Effect of the dye concentration in feed solution on the dye removal efficiency (R%) of the membrane prepared at different GO concentrations.

Figure 9 illustrates the dye removal efficiency (%R) of the control PES and PES–PVP–GO membranes for a 50-ppm dye concentration in simulated wastewater, pH = 5, and feed solution temperature of 25 ± 1 °C. The removal efficiency (%R) of the Acid Black dye was 88% for the control PES membrane, which highly improved to 99% for the membrane prepared from 0.1 wt% GO. The removal efficiency was between 99.4% and 99.7% for any further increase in the contents of GO. Whereas the removal efficiency of the control PES membrane for the Rose Bengal dye was 86%, it improved to 96.2% with an addition of 0.1 wt% GO. Using 0.2 wt% GO in the casting solution increased the removal efficiency to 97%, while further increases in the GO amounts (e.g., 0.3, 0.5, 1.5, and 2%) resulted in an improvement in the removal efficiency of between 98.9% and 99%, as depicted in Figure 9. For both types of dyes, the removal efficiency (%R) of the PES–PVP–GO membranes was substantially better than that of the membrane fabricated from the control PES membrane.

The negative hydrophilic functional groups on the GO surface, such as hydroxyl (-OH) and carboxyl (-COOH), can create a high zeta potential by generating negative charges on the membrane surface. The negative charge of the Acid Black dye and the negative charge on the membrane surface will increase the repulsive force, which leads to an increase in the rejection rate of the dye [54]. The control PES membrane does not function in this manner. Fundamentally speaking, the charge that repels the dye depends on the membrane charge, ionic strength, and ionic capacity [45], which is due to the greater repulsion between the dye and the functional groups of the embedded nanomaterial. In addition, some researchers have proposed that due to a strong bond with water, a modified hydrophilic membrane can effectively prevent molecules from passing through [55]. 

#### 3.2.2. Integrity and Stability of the PES Membranes during Long-Term Performance

**Figure 10:**
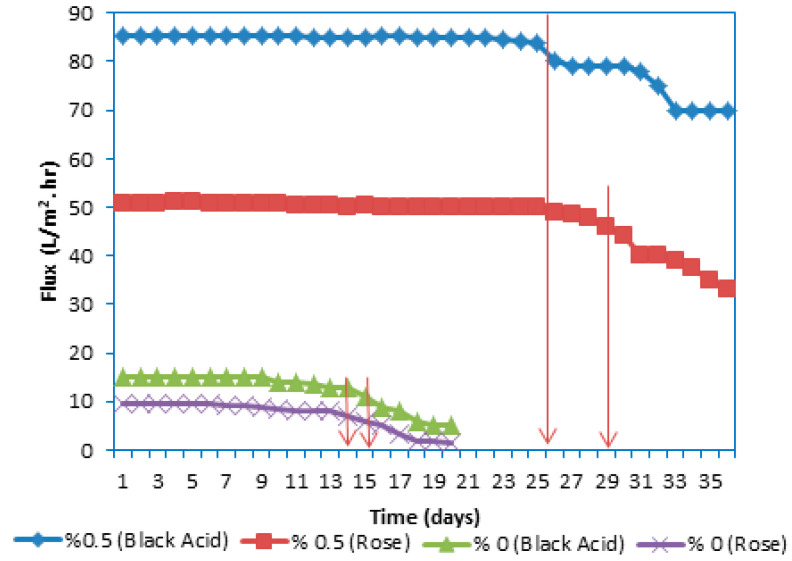
The effect of GO in casting solution on long-term of the prepared membrane.

Under extreme conditions (such as pressure and temperature), periodic exposure of the membrane to the cleaning agent will negatively affect the physical properties of the membrane, which in turn may affect the performance of the membrane. As such, the quality and quantity of products may be affected. In addition, in a cross-flow system, contamination may occur in the membrane module, and the contamination cannot be effectively removed through an organized process. Therefore, it is often necessary to study the life of the reusable membrane to ensure that the membrane will not collapse over time during the filtration process, where it can affect the products and even the process itself [14]. Therefore, this work also investigated the long-term consequences of processing two dyes using membranes fabricated from the control PES and PES–PVP–GO with 0.5 wt% GO, finding that the GO/PES membrane provided optimal performance. Figure 10 depicts the long-term UF operation carried out for the 0.5 wt% GO (GO/PES) membrane at a pressure of 3 bar and feed temperature of 25 °C for 50-ppm Acid Black and Rose Bengal dye solutions. Figure 10 demonstrates that the permeation flux of the Acid Black solution using the control PES membrane was stable between 13 and 15.1 L/m^2^·h throughout the UF operation for 14 d before the permeation flux began to decline. In contrast, the long-term operation of the membrane fabricated from 0.5 wt% GO using an Acid Black dye solution improved greatly over 26 d, and the permeation flux remained stable between 85.2 and 80 L/m^2^ h.

For the Rose Bengal dye solution, the permeation flux of the control PES membrane was stable throughout the UF operation between 9.7 and 8 L/m^2^ h for 14 d. Additionally, the long-term operation of the membrane fabricated from 0.5 wt% GO using the Rose Bengal dye solution was highly enhanced by 26 d, and the permeation flux remained stable between 50.8 and 49 L/m^2^ h. The hydrophilic nature of the GO particle was the main reason for the permeate flux stability of this membrane. However, the results of the long-term performance test indicate the feasibility of using GO as an additive in the GO/PES membrane for efficient dye removal from wastewater treatment using the UF process. The main reason for long and stable performance during the long-term UF operation was the GO embedded in the membrane matrix, where the GO acted as an effective antifouling material, which in turn protected the thickness of the membrane wall. Additionally, the reason for the stable performance was attributed to the excellent mechanical properties of GO, as was discussed previously in Section 3.1.6. “Mechanical properties (tensile strength)”.

#### 3.2.3. Flux Recovery Efficiency

**Figure 11:**
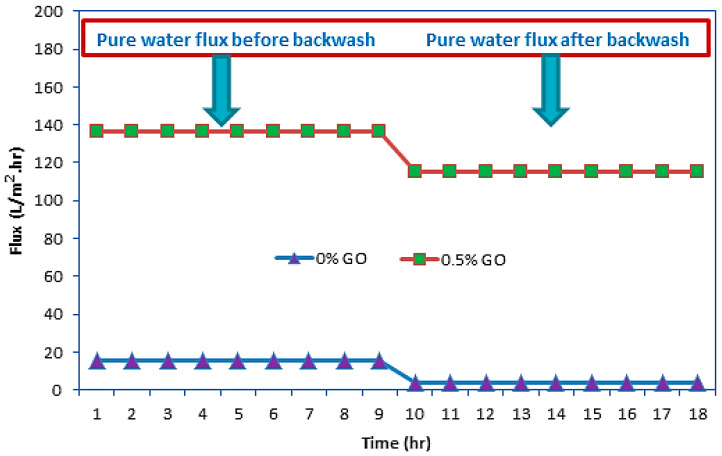
Pure water flux before and after backwashing for 18 h at pressure of 3 bar.

The membrane used in the ultrafiltration system should be able to restore its water permeability after contact with wastewater. Therefore, it is necessary to evaluate and compare the flux before and after ultrafiltration. After completing the long-term performance experiment, the flat sheet PES-UF membrane was backwashed with pure water for 9 h at 3 bar, after which the pure water flux was measured again (see Figure 11). Figure 11 shows that the water flux after backwashing was lower than that in Figure 7, which was caused by the dye and contaminants remaining on the surface of each membrane. This phenomenon will cause a sudden drop in flux during long-term operation. In contrast, for the 0.5 wt% GO concentration, the adsorption effect between the dye pollutant and the membrane was very weak because of the beneficial hydrophilic property of the membrane; therefore, the dye can be easily washed away from the membrane surface containing the GO particles after the backwash. Finally, the membrane surfaces returned to being white in color, and the pure water flux recovered.

#### 3.2.4. Effect of the Feed pH on the Permeation Flux and Dye Removal

**Figure 12:**
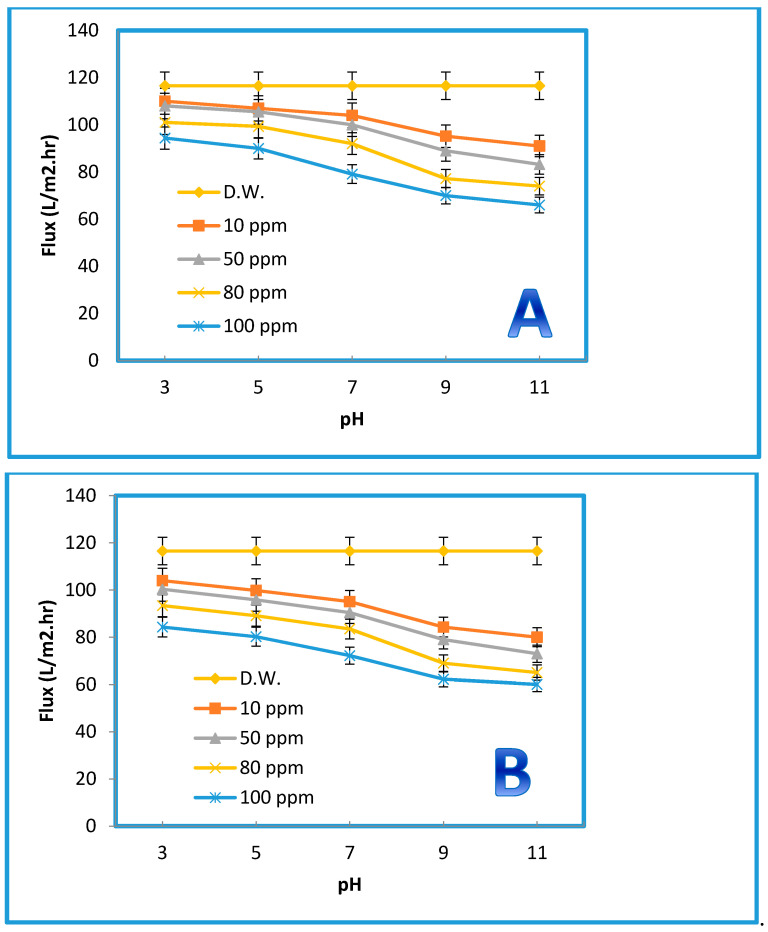
Effect of pH on the permeation flux of membrane prepared from % 0.5 GO, at 3 bar, 25 °C, for Acid Black (AB-210) (**A**) and Rose Bengal (RB) (**B**).

**Figure 13:**
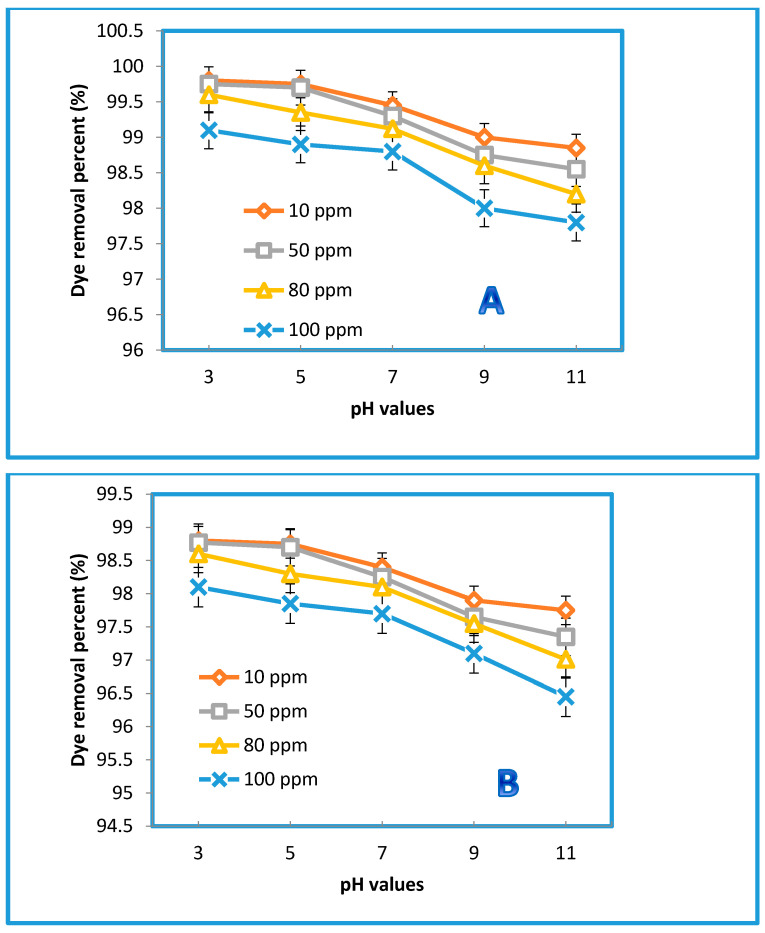
Effect of pH on the dye removal percent of membrane prepared from % 0.5 GO, at 3 bar, 25 °C, for AB-210 (**A**) and RB (**B**).

Changing the zeta potential by changing the pH of the solution can critically influence the performance of the membrane separation. Measurements of zeta potential are not only essential for obtaining insight into the retention mechanisms for disparate charged solutes but also for understanding membrane aging, fouling, functionalization, and cleaning. Changing the pH value will not only dissociate the surface functional groups but also induce the membrane to acquire a charge from the adsorbed molecules in the feed [56]. The pH of the feed solution could influence the degree of ionization and speciation of the dyes [57]. A wide range of pH values (from 3 to 11) was employed to examine the membranes’ performance behavior in terms of their permeate flux against both dyes. As displayed in Figure 12, there was a clear influence of the pH on the permeate flux of the blended membrane prepared from 0.5% GO (i.e., the optimum concentration). However, a slight decrease in the permeation flux was observed at pH = 7, while beyond this value at a higher pH, the decline of the permeation flux became more significant for both dyes at all concentrations (i.e., 10, 50, 80, and 100 ppm). Interestingly, the pure water flux did not manifest any change, indicating that PES membranes can tolerate a wide pH range. Additionally, a higher flux decline was observed at higher concentrations (e.g., 100 ppm). This was probably caused by the dissociation of the functional groups of the organic dyes, along with the dissociation of the membrane surface. Indeed, reducing the interactions between the membrane surface and these molecules would have accelerated their deposition on the surface. The removal rates were calculated for both dyes at the optimum concentration (0.5% GO) at different pH levels. Figure 13 illustrates the impact of 0.5 wt% GO on the removal % of both dyes. As shown in Figure 13, different concentrations of the AB-210 and RB dyes were used. The rate of dyes removal decreases gradually by increasing the concentration of the dye as well as for the relationship between pH with the rate of removal where the rate of dyes removal decreases significantly by increasing the value of pH (basic condition). Additionally, the dye removal % for Rose Bengal was lower than the dye removal % for Acid Black-210 because the molecular weight of Rose Bengal (1017 g/mol) is slightly higher than that of AB-210 (938.017 g/mol) [58,59].

**Figure 14:**
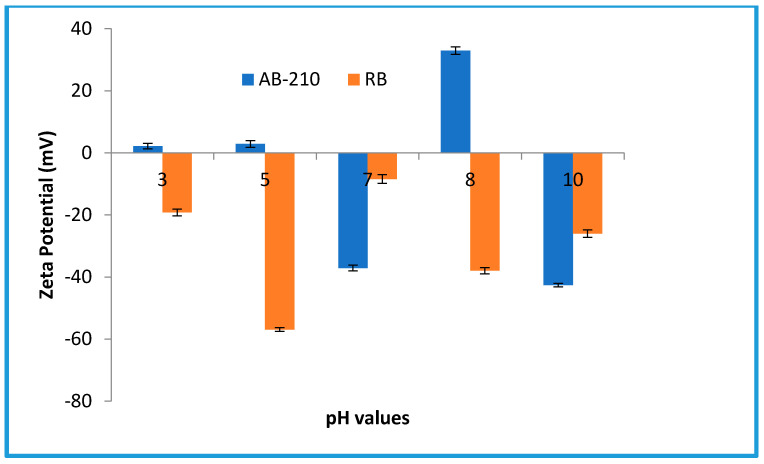
Dependence of zeta potential of dye solutions on pH value.

Moreover, according to the zeta potential measurements displayed in Figure 14, the two dyes possess negative surface energy. Therefore, the two anionic AB-210 and Rose Bengal dyes showed less degradation due to the lower binding affinity caused by their negative charge. Furthermore, because of the acidic functional groups of GO, it can prompt negative charge on the membrane composite surface, causing high rejection between negative surface and negative dye.

To highlight the novelty of this work, a comprehensive comparison of membrane separation performance with other reported/published data is presented in Table 3. This comparison was made with the recent reported data found in the literature based on permeation water flux (PWF) and the solute rejection rate (%R) of GO mixed matrix membranes (MMMs). Relatively few polymers combined with different concentrations of GO-NPs have been recently reported for ultrafiltration applications. From Table 3, it can be distinguished that the membranes synthesized in the present work, with the operating parameters discussed in Section 2.3. “Preparation of the membrane” from GO mixed matrix membranes (MMMs), have acceptable permeation water flux and higher rejection of contaminates.

## 4. Conclusions

In the present work, GO nanoparticles were successfully synthesized and then applied to modify PES and PVP UF membranes without the agglomeration of GO. Different amounts of GO particles (i.e., 0.1, 0.2, 0.3, 0.5, 1.5, and 2 wt%) were added to the casting solution during the phase inversion process to obtain modified composite membranes, and PES/GO flat-sheet membranes were prepared and used to remove dyes from simulated leather tanning and textile industries’ wastewater.

Adding GO particles to the PES casting solution changed the morphological structure of the membrane and significantly increased the mean pore radius. Due to the increase in the hydrophilicity of the particles, the pure water permeability (PWP) of the membrane increased after the addition of the GO particles. The CA value of the top surface decreased from 60.82° of the control PES to 39.21° when the content of the GO particles increased to 0.5 wt%. This is due to the GO particles moving towards the surface during the membrane formation process, which in turn makes the surface more hydrophilic. Therefore, the mechanical strength of the composite membrane can be slightly improved, which can be attributed to the reinforcing effect of the inorganic filler. The tensile strength of the PES membrane increased by about 69% after the addition of 0.5 wt% of GO-NPs to the casting solution in comparison to the control PES membrane.

The pore structures and the negative charge of the nanomaterial led to an increase in the permeability to about 88% at the optimal amount of 0.5 wt% GO nanomaterial. Additionally, the addition of GO to the PES casting solution resulted in longer lifetimes of the membranes due to enhancement in the fouling resistance and flux recovery efficiency (FRE) after backwashing.

The dye removal was higher than 99% for all the membranes studied and both dyes (AB-210 and RB), at dye concentrations of 10, 50, 80, and 100 ppm and an operating pressure of 3 bar. These conditions can be utilized in the decolorization and dye purification of wastewater.

## Figures and Tables

**Table 1:** The compositions of the casting solution.

Membrane Label	PES Content (wt.%)	PVP Content (wt.%)	DMSO Content (wt.%)	GO Content (wt.%)
1	21	1	78	0
2	21	1	78	0.1
3	21	1	78	0.2
4	21	1	78	0.3
5	21	1	78	0.5
6	21	1	78	1.5
7	21	1	78	2

**Table 2:** Mean surface pore size and roughness for the top surface of the blend membranes.

Percent of GO in PES Membrane	Top Surface Mean Pore Radius (nm)	Mean Roughness (nm)
0	8.25	45.5
0.1	5.29	36
0.2	11.55	111
0.3	14.06	77.1
0.5	14.59	22
1.5	15.59	41
2	15.22	28.9

**Table 3:** Demonstrates the comparison of this work with the recent published GO mixed matrix membranes.

Polymer (wt.%)	Fillers (wt.%)	Pore-Formers PVP (wt.%)	Permeate (LMH bar)	Rejection (%)	Ref.
PES (16)	* Cu(TPA)@GO	PVP (2)	24–75	15% Methyl blue 65% Methyl orange 90% Congo red	[32]
PES (18)	sulfonated-GO (1)	PVP (1)	9.1	83.9% Acid blue 83.5% Bismark Brown	[33]
PES (20)	GO (0.3)	T904 (5)	245	62.3% sunset yellow (SY) dye 35.4% acridine orange (AO) dye	[34]
PES (18)	GO (0.008)	PVP (1)	289.86	86.58% methyl red	[35]
PES (16)	* APTS-GO (0.1)	PVP-40 (1)	9.9	97.4% SY 96.5% AO 51.6% MgSO_4_	[36]
PSf (16)	GO (0–5)	No additive used	87.5–150	No rejection data	[37]
PSf (15)	Janus GO (0.1–1)	No additive used	11.5–115	92% BSA	[38]
PVDF (15)	GO (0–2)	PVP (1)	188.36	52% BSA	[39]
PVDF (12)	GO (0.5, 1 and 2%)	PVP (5)	552.92	87.11% COD 93.75% NH_4_-N	[40]
PES (21)	GO (0.1–2) wt.%	PVP (1)	116.5	99.7% Acid black (AB) dye 99% Rose Bengal dye (RB)	This work

* APTS is defined as aminopropyltriethoxysilane; TPA is defined as Terephthalate.

## Supplementary Materials

The following are available online at https://www.mdpi.com/2077-0375/10/12/366/s1.
