# Supplementary material for: Removal of Dyes Using Graphene Oxide (GO) Mixed Matrix Membranes
Source: Membranes (Basel). 2020 Nov 25;10(12):366. doi: 10.3390/membranes10120366 (PMC7760904; doi:10.3390/membranes10120366)
Supplement: Supplementary file 1 [file membranes-10-00366-s001.pdf]

membranes

Supporting Information (SI)

Removal of dyes using graphene oxide mixed matrix membranes

Rana J. Kadhim [1], Faris H. Al-Ani [1], Muayad Al-shaeli [2], Qusay F. Alsalhy [3,*] and Alberto Figoli [4]

[1] Civil Engineering Department, University of Technology, Alsinaa Street 52, Baghdad 10066, Iraq; rana1979.kadhim@gmail.com (R.J.K.); 40027@uotechnology.edu.iq (F.H.A.-A.)

[2] Department of Chemical Engineering, Monash University, Clayton VIC 3800, Australia; muayad.al-shaeli@monash.edu (M.A.)

[3] Membrane Technology Research Unit, Chemical Engineering Department, University of Technology, Alsinaa Street 52, Baghdad 10066, Iraq

[4] Institute on Membrane Technology, National Research Council (ITM-CNR), 87030 Rende, Italy; a.figoli@itm.cnr.it

* Correspondence: qusay_alsalhy@yahoo.com or 80006@uotechnology.edu.iq; Tel.: +964-790-173-0181

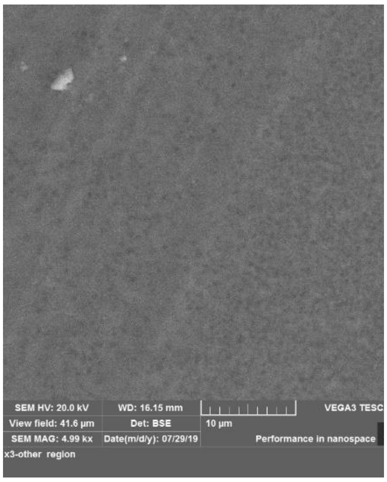

% 0 GO

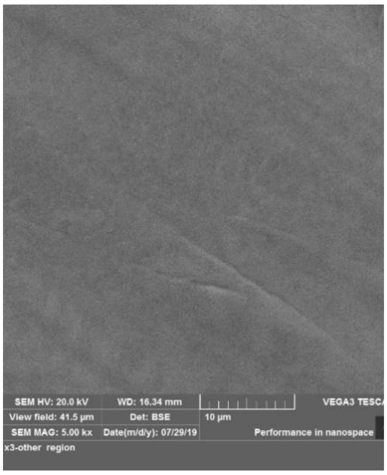

% 0.1 GO

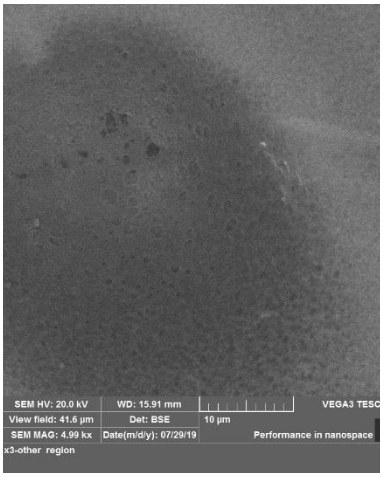

% 0.2 GO

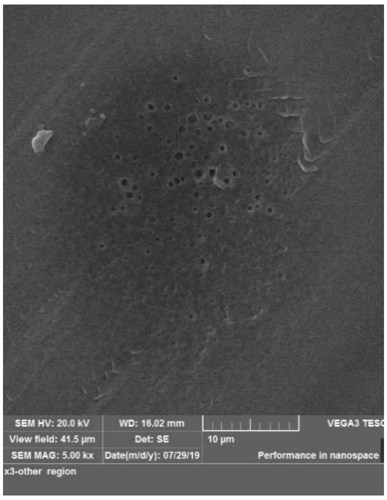

% 0.3 GO

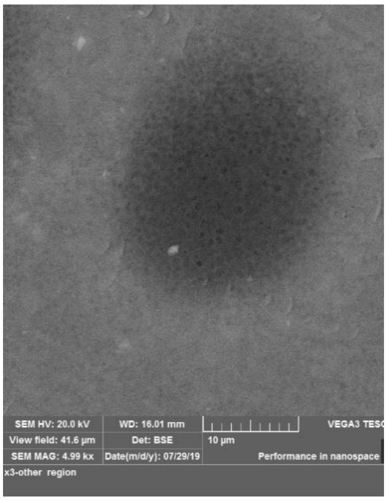

% 0.5 GO

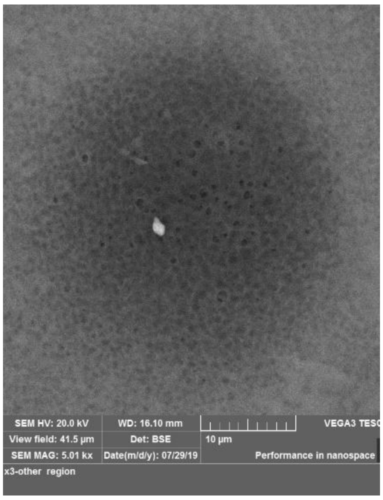

% 1.5 GO

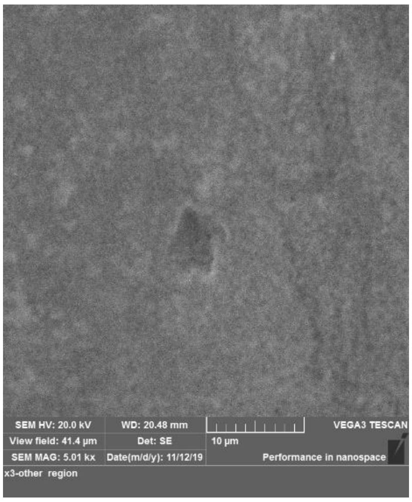

% 2 GO

Figure S1. Top surface SEM images of control and GO modified PES membranes.

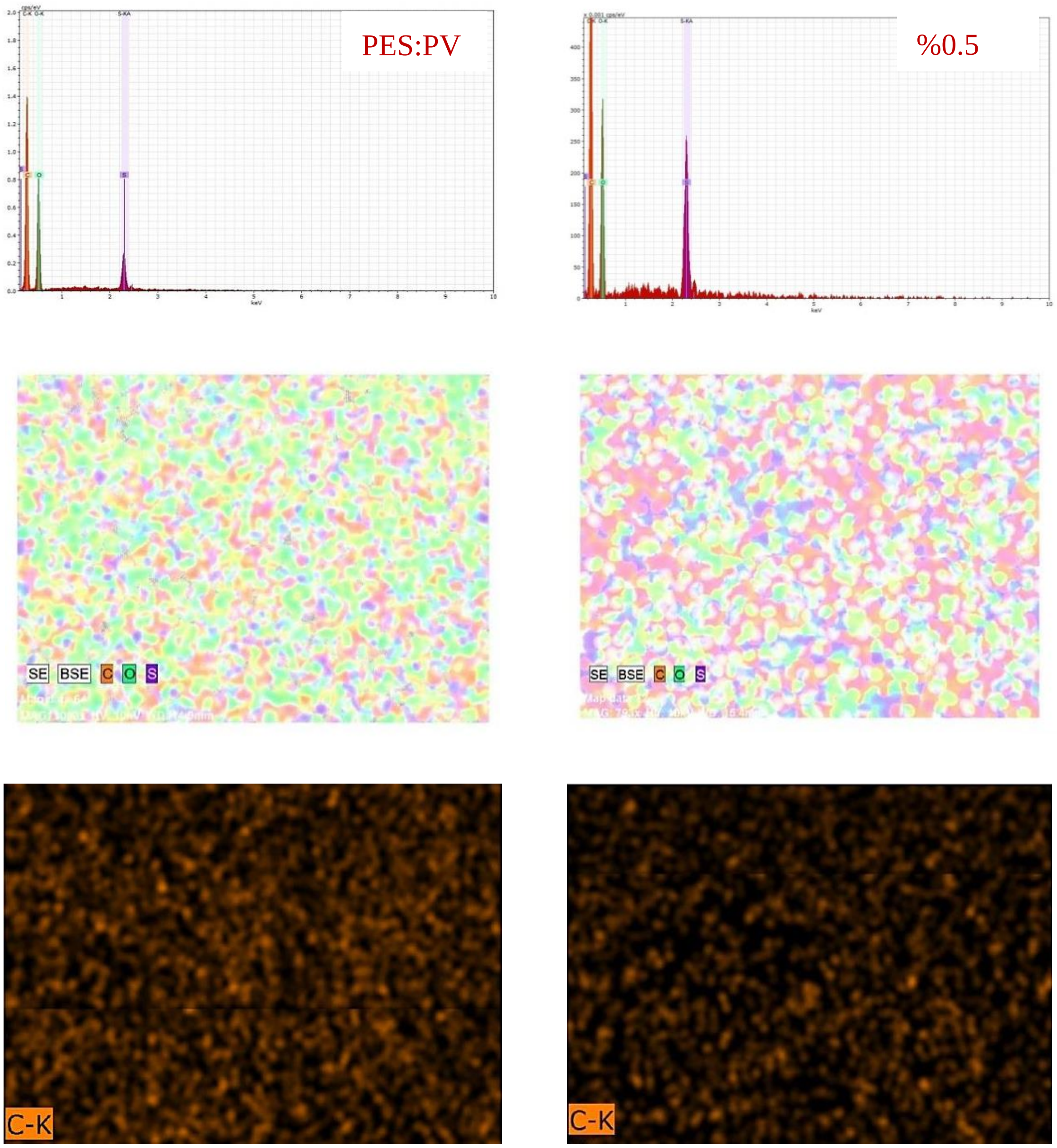

% 0 GO **% 0.5 GO**

Figure S2. EDX for control and GO modified PES membranes was verified by the EDX analysis.

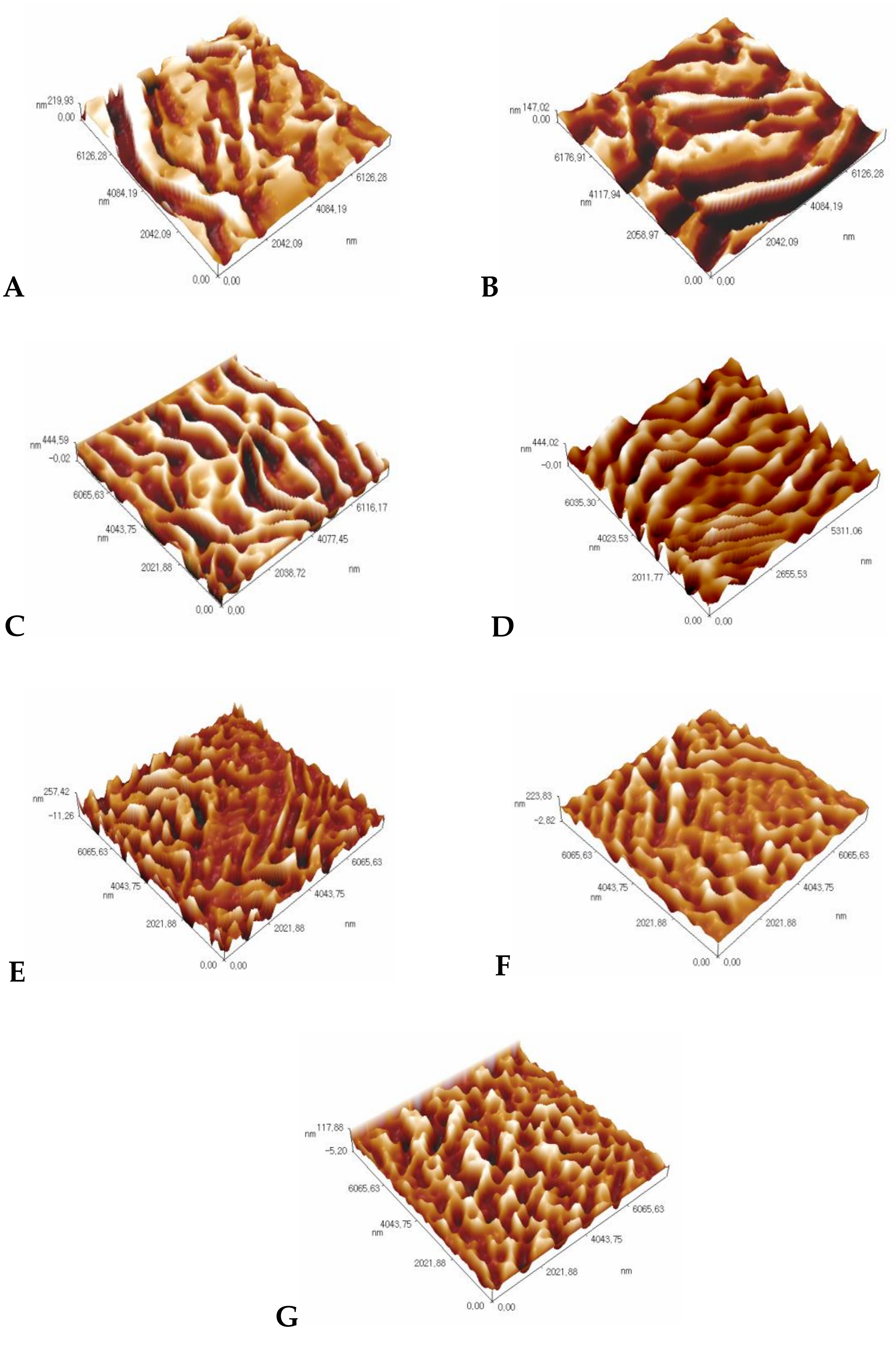

Figure S3. 3D-AFM images of the modified and unmodified membranes, top surface (**A**) % 0 GO, (**B**) % 0.1 GO, (**C**) % 0.2GO, (**D**) % 0.3 GO, (**E**) % 0.5 GO, (**F**) % 1.5 GO, (**G**) % 2 GO
